# High ROS Production by Celecoxib and Enhanced Sensitivity for Death Ligand-Induced Apoptosis in Cutaneous SCC Cell Lines

**DOI:** 10.3390/ijms22073622

**Published:** 2021-03-31

**Authors:** Jiaqi Zhu, Stefanie May, Claas Ulrich, Eggert Stockfleth, Jürgen Eberle

**Affiliations:** 1 Department of Dermatology, Venerology and Allergology, Skin Cancer Center, Charité Universitätsmedizin Berlin, 10117 Berlin, Germany; zhujiaqijiaqi@gmail.com (J.Z.); stefanie.may@charite.de (S.M.); Claas.Ulrich@charite.de (C.U.); 2 Department of Gynecology and Obstetrics, Jilin University, Changchun 130012, China; 3 Department of Dermatologie, Venerologie und Allergologie, Klinikum Bochum, Ruhr-Universität Bochum, 44791 Bochum, Germany; e.stockfleth@klinikum-bochum.de

**Keywords:** cutaneous SCC, celecoxib, apoptosis, reactive oxygen species, TRAIL, death ligands

## Abstract

Incidence of cutaneous squamous cell carcinoma (cSCC) and actinic keratosis has increased worldwide, and non-steroidal anti-inflammatory drugs as celecoxib are considered for treatment. We show here strong anti-proliferative effects of celecoxib in four cSCC cell lines, while apoptosis and cell viability largely remained unaffected. Impeded apoptosis was overcome in combinations with agonistic CD95 antibody or TNF-related apoptosis-inducing ligand (TRAIL), resulting in up to 60% apoptosis and almost complete loss of cell viability. Proapoptotic caspase cascades were activated, and apoptosis was suppressed by caspase inhibition. TRAIL receptor (DR5) and proapoptotic Bcl-2 proteins (Puma and Bad) were upregulated, while anti-apoptotic factors (survivin, XIAP, cFLIP, Mcl-1, and Bcl-w) were downregulated. Strongly elevated levels of reactive oxygen species (ROS) turned out as particularly characteristic for celecoxib, appearing already after 2 h. ROS production alone was not sufficient for apoptosis induction but may play a critical role in sensitizing cancer cells for apoptosis and therapy. Thus, the full therapeutic potential of celecoxib may be better used in combinations with death ligands. Furthermore, the immune response against cSCC/AK may be improved by celecoxib, and combinations with checkpoint inhibitors, recently approved for the treatment of cSCC, may be considered.

## 1. Introduction

Actinic keratosis (AK) is derived from neoplastic epidermal keratinocytes and is characterized by high prevalence and the risk to proceed into invasive cutaneous squamous cell carcinoma (cSCC). About 20% of skin cancer deaths worldwide are related to cSCC [1]. Thus, it is the second most common skin cancer in Caucasians and East Asians (including Japanese and Chinese) and even the most common skin cancer in African Americans [2,3].

The effects of anticancer drugs are strongly related to the induction of apoptosis, a mechanism basic for the control of tissue homeostasis [4]. Thus, resistance to apoptosis represents a crucial step in oncogenesis and drug resistance [5]. Intrinsic proapoptotic pathways are activated in response to cellular stress situations as well as by anticancer treatment, e.g., by chemotherapy. This relies on a loss of mitochondrial membrane potential and the release of mitochondrial factors such as cytochrome c, which triggers activation of initiator caspase-9 [6].

On the other hand, extrinsic induction of apoptosis is initiated by death ligands such as CD95L/FasL and TRAIL (TNF-related apoptosis-inducing ligand). Upon death receptor activation, death-inducing signaling complexes are formed, resulting in activation of initiator caspases, such as caspase-8 and caspase-10 [7]. Initiator caspases may cleave and thus activate effector caspases as -3, -6, and -7, which cleave a large number of death substrates with the final result of DNA fragmentation and apoptosis induction [8]. In particular, the death ligand TRAIL has attracted much consideration due to its anticancer activity, while normal cells are largely spared [9,10]. However, cancer cells may also develop TRAIL resistance, limiting its clinical applicability [11].

Besides the well-established pathways, there is increasing evidence for a particular role of reactive oxygen species (ROS) in the control of apoptosis. In melanoma as well as in cutaneous T-cell lymphoma cells, we have previously described enhanced ROS levels after inhibition of phosphoinositol-3 kinase and BRAF as well as in response to indirubin derivatives [12,13,14,15]. However, the relation of ROS to previously described apoptosis pathways remained largely fragmentary so far.

Cyclooxygenase-2 (COX-2) represents the primary cancer target of NSAIDs (nonsteroidal anti-inflammatory drugs) [16]. The NSAID and COX-2 inhibitor celecoxib has attracted broad attention because of its antitumor activities. Thus, clinical trials have shown efficiency in colorectal adenoma, leading to FDA approval of celecoxib for patients with familial adenomatous polyposis [17,18,19]. Concerning COX-2, however, increasing evidence in the last years has shown that the anticancer activity of celecoxib may be more related to COX-2-independent effects [20,21,22].

Drug combinations may overcome the problems of limited efficiency and resistance, but the combination of celecoxib and TRAIL was not investigated in cSCC so far. Celecoxib’s mode of action in cSCC also remained largely elusive. Here, we show in cSCC cells that apoptosis by celecoxib can be strongly enhanced in combinations with TRAIL or with CD95 agonistic antibodies. This was related to caspase activation as well as to the regulation of apoptosis-related proteins. Finally, enhanced levels of reactive oxygen species (ROS) appeared as highly characteristic for celecoxib, although ROS levels alone were not sufficient for apoptosis induction. The results may be considered for improving cSCC/AK therapy.

## 2. Results

### 2.1. Decreased Cell Proliferation by Celecoxib Despite Little Effect on Apoptosis

**Figure 1:**
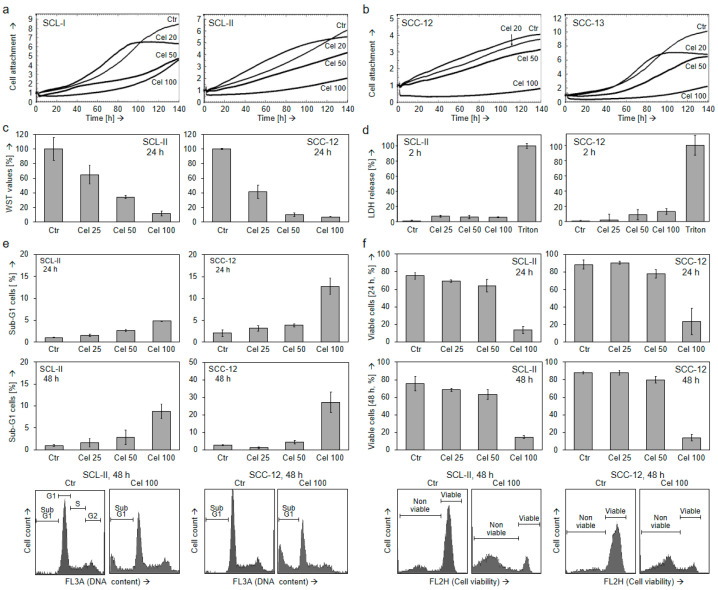
Decreased cell proliferation by celecoxib despite little effects on apoptosis. (**a**,**b**) Real-time cell analysis (RTCA) of (**a**) squamous cell carcinoma cell lines SCL-I and SCL-II as well as (**b**) of squamous cell carcinoma cell lines SCC-12 and SCC-13 treated with 20, 50, and 100 µM celecoxib (time 0 h = start of treatment). The determined cell index gives a relative measurement of cell attachment. The experiment was performed twice with triple values, which revealed highly comparable results. (**c**) Cell proliferation was quantified by WST-1 assay in SCL-II and SCC-12 at 24 h of celecoxib treatment (25, 50, 100 µM). (**d**) Direct cytotoxicity was determined in SCL-II and SCC-12 at 2 h of treatment by quantification of lactate dehydrogenase (LDH)-release (fold change of non-treated controls, set to 1). Cells treated with triton x100 (0.7%) were used as positive controls. (**e**,**f**) Apoptosis (**e**) and cell viability (**f**) were determined in SCL-II and SCC-12 at 24 h and at 48 h in response to 25, 50, and 100 µM celecoxib. Apoptotic cells were identified in cell cycle analyses as sub-G1 cells characterized by DNA fragmentation, whereas cell viability was determined by calcein-AM staining. Examples of flow cytometry measurements are given below. (**c**–**f**) Mean values are shown in representative experiments. At least two independent experiments, each one with triplicates, revealed highly comparable results.

We evaluated the therapeutic potential of celecoxib in four representative cSCC cell lines (SCL-I, SCL-II, SCC-12, and SCC-13). Real-time cell analysis (RTCA) showed a dose-dependent decrease in cell proliferation in response to celecoxib (20, 50, and 100 µM), which started almost immediately after the onset of treatment (time: 0; Figure 1a,b). In SCL-II and SCC-12, an additional WST-1 assay was performed at 24 h and proved dose-dependent anti-proliferative effects with revealed values of 35% (SCL-II) and 10% (SCC-12) for 50 µM celecoxib (Figure 1c). Anti-proliferative effects were not based on direct cell cytotoxicity, as determined by lactate dehydrogenase (LDH) release assays in SCL-II and SCC-12 at 2 h after the start of celecoxib treatment (Figure 1d).

In contrast, apoptosis induction was much less pronounced and below 5% for treatments with 25 and 50 µM celecoxib at 24 and 48 h (Figure 1e). Similarly, only little effects were obtained at the level of cell viability for 25 and 50 µM (9–16% reduction, as compared to controls). Somewhat stronger effects were seen only for 100 µM celecoxib (73–84% reduction, as compared to controls; Figure 1f). Thus, while cell proliferation was clearly affected also by moderate celecoxib concentrations (25 and 50 µM), direct effects on apoptosis and cell viability were limited.

### 2.2. Strongly Enhanced Apoptosis in Combinations with Death Ligands

**Figure 2:**
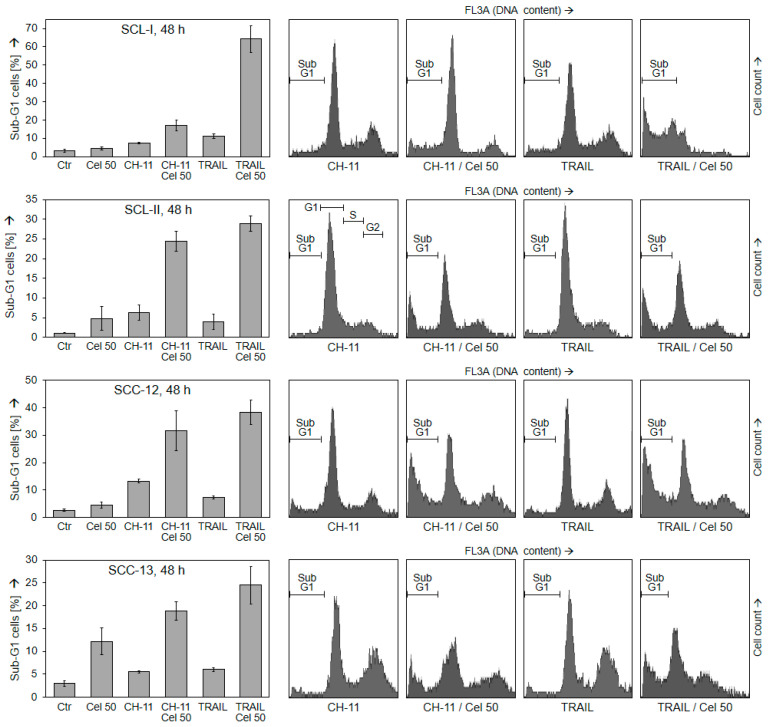
Induction of apoptosis in combinations with death ligands. Cell lines SCL-I, SCL-II, SCC-12 and SCC-13 were treated with celecoxib (50 µM), CH-11 (100 ng/mL), TRAIL (50 ng/mL) or combinations. Apoptosis was determined at 48 h by propidium iodide staining and cell cycle analysis. Left, results of representative experiments are shown. At least two independent experiments, each one with triplicate values, revealed highly comparable results. Right side, examples of flow cytometry measurements are shown (CH-11, TRAIL and combination treatments); apoptotic cell populations (sub-G1) are indicated.

In order to identify conditions that may increase the proapoptotic effects of celecoxib, it was combined with TRAIL and CD95 agonistic antibody CH-11. This revealed strongly enhanced apoptosis in all cell lines. Thus, the combination of celecoxib (50 µM) with TRAIL (50 ng/mL) for 48 h resulted in apoptosis rates of 64%, 29%, 38%, and 25% in SCL-I, SCL-II, SCC-12, and SCC-13, respectively. Similarly, the combination with CH-11 (100 ng/mL) resulted in apoptosis rates of 17%, 24%, 32%, and 19% (Figure 2).

**Figure 3:**
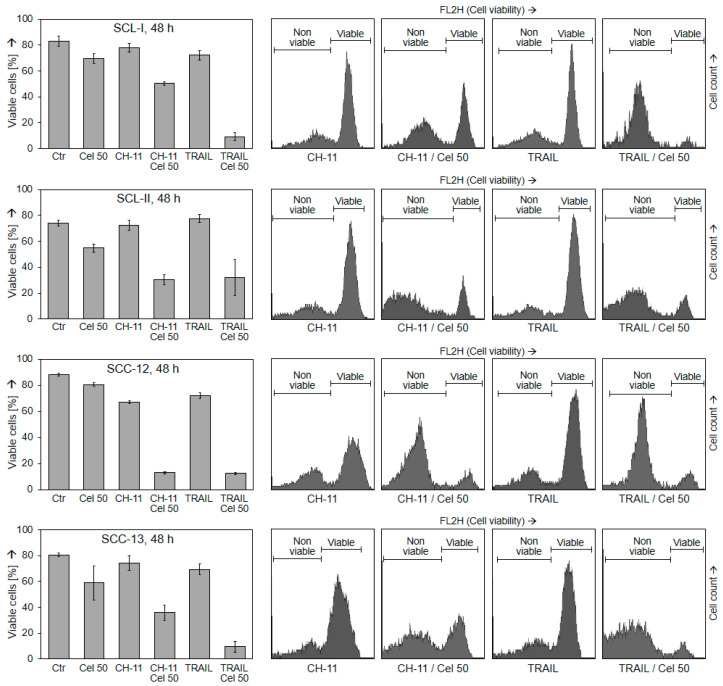
Loss of cell viability in combinations with death ligands. Cell lines SCL-I, SCL-II, SCC-12 and SCC-13 were treated with celecoxib (50 µM), CH-11 (100 ng/mL), TRAIL (50 ng/mL) or combinations. Cell viability was determined at 48 h by calcein staining and flow cytometry. Left, results of representative experiments are shown. At least two independent experiments, each one with triplicate values, revealed highly comparable results. Right side, examples of flow cytometry measurements (CH-11, TRAIL and combination treatments) are shown; viable and non-viable cell populations are indicated.

Enhanced apoptosis came along with a reciprocal loss of cell viability. Thus, in combinations of celecoxib with TRAIL, cell viability at 48 h was decreased to 9%, 32%, 13%, and 10% in SCL-I, SCL-II, SCC-12, and SCC-13, respectively. In combination with CH-11, it was decreased to 50%, 30%, 13%, and 36%, respectively (Figure 3). Thus, death ligands and celecoxib appeared as highly effective combination partners.

### 2.3. Changes of Mitochondrial Membrane Potential

**Figure 4:**
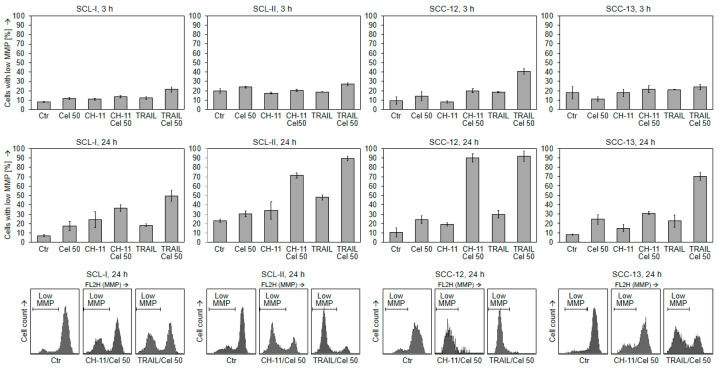
Changes of mitochondrial membrane potential (MMP). Four cSCC cell lines were treated with celecoxib (50 µM), CH-11 (100 ng/mL), TRAIL (50 ng/mL) or combinations for 3 h and 24 h, respectively. Percent of cells with low MMP were determined by TMRM^+^ staining. Mean values and SDs of representative experiments are shown. At least two independent experiments, each one consisting of triplicate values, revealed highly comparable results. Example cytometry measurements of controls and combination treatments are given below; cell fractions with low MMP are indicated.

Addressing the mechanisms of celecoxib-mediated effects in cSCC cells, we determined changes in the mitochondrial membrane potential (MMP). No major changes of MMP were seen at early times (3 h) in response to celecoxib or combination treatment, clearly not suggestive for the initial activation of intrinsic apoptosis pathways. At 24 h, celecoxib treatment alone also did not result in strong effects at the level of MMP (<30% cells with low MMP). Significant loss of MMP (up to 90%) was seen only in combination when apoptosis was also strongly induced (Figure 4). Thus, loss of MMP appeared in parallel with induced apoptosis or as a result of it.

### 2.4. Enhanced Caspase Activation in Course of Combined Treatment

**Figure 5:**
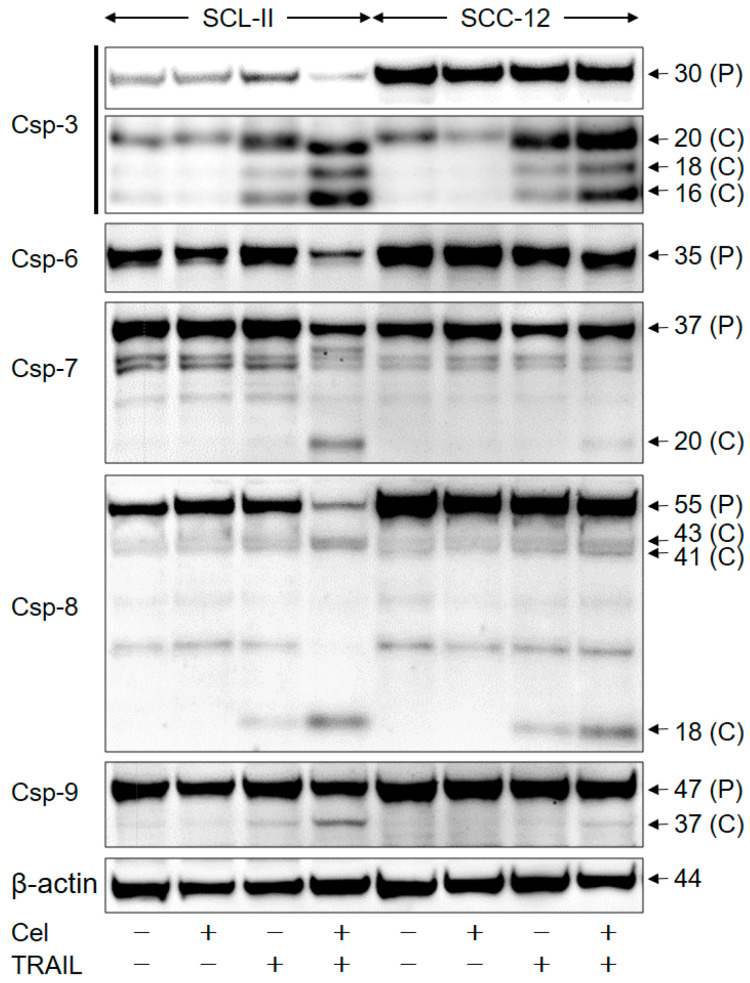
Enhanced caspase activation in the course of combination treatment. SCL-II and SCC-12 cells were treated for 24 h with celecoxib (50 µM) and/or TRAIL (50 ng/mL). Protein extracts were analyzed by Western blotting for expression of caspase-3, caspase-6, caspase-7, caspase-8 and caspase-9. Equal protein amounts (30 µg per lane) were separated by SDS-PAGE, and consistent blotting was proven by Ponceau staining as well as by evaluation of expression of β-actin. Proforms (P) as well as caspase cleavage products (C) were identified. Two independent series of protein extracts and independent Western blots revealed highly comparable results.

To distinguish between caspase-dependent and independent mechanisms, caspase activation in response to celecoxib and TRAIL was investigated in SCL-II and SCC-12 by Western blotting. This included effector caspases -3, -6, and -7, as well as caspase-8 and caspase-9, the major initiator caspases of the extrinsic and intrinsic apoptosis pathways. While TRAIL alone resulted in some caspase processing, in particular of caspase-8 (43, 41, and 18 kDa) and caspase-3 (20, 18, and 16 kDa), celecoxib alone remained completely without effect on caspase activation (no processing) (Figure 5).

Activation of caspase-8 and caspase-3 were strongly enhanced in the combination of celecoxib/TRAIL. Similarly enhanced was the activation of caspase-6 (reduction in the 35 kDa proform), caspase-7 (reduction in the 37 kDa proform and cleavage products at 20 kDa), and caspase-9 (cleavage products of 37 kDa) (Figure 5). Collectively, these data indicate a complete activation of proapoptotic caspase cascades by combination treatment.

**Figure 6:**
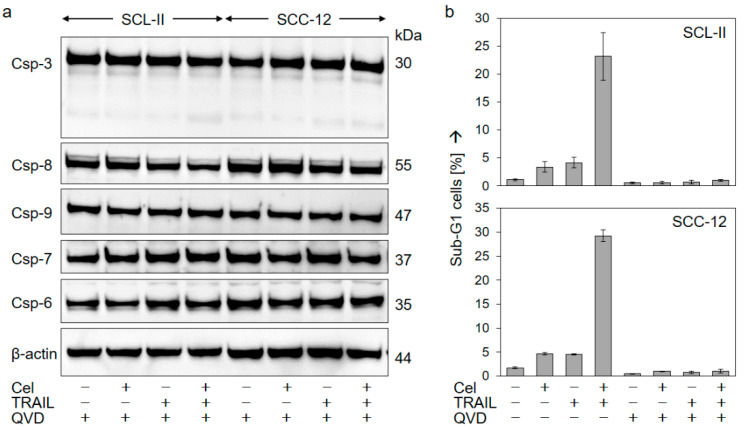
Inhibition of apoptosis by a caspase inhibitor. SCL-II and SCC-12 were treated for 24 h with celecoxib (50 µM), TRAIL (50 ng/mL), or the combination. In addition, cells received the pan-caspase inhibitor QVD-Oph (10 µM) at 1 h before other treatments started. (**a**) Protein extracts were analyzed by Western blotting for caspase activation (processing). Equal protein amounts (30 µg per lane) were separated by SDS-PAGE, and consistent blotting was proven by Ponceau staining as well as by evaluation of β-actin expression. Molecular weights (in kDa) of caspase proforms are indicated. Two independent series of protein extracts and independent Western blots revealed highly comparable results. (**b**) Apoptosis was determined at 24 h of treatment by cell cycle analysis and quantification of sub-G1 cells (mean values +/− SDs of representative experiments). Two independent experiments, each one with triplicate values, showed the same result.

The pan-caspase inhibitor QVD-Oph was applied to prove the decisive role of caspases. In SCL-II and SCC-12, QVD-Oph completely abrogated any caspase processing (Figure 6a) as well as apoptosis induction (Figure 6b), thus proving the significant role of caspases in this setting.

### 2.5. Regulation of Mediators of Apoptosis and Cell Proliferation

**Figure 7:**
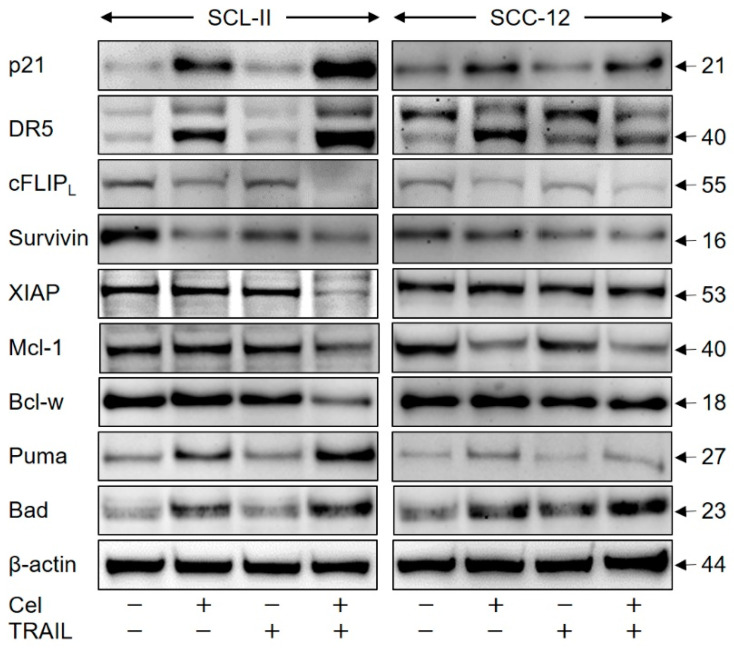
Regulation of characteristic mediators of apoptosis and cell proliferation. Cell lines SCL-II and SCC-12 were treated for 24 h with celecoxib (50 µM), TRAIL (50 ng/mL) or the combination. Expression levels of p21 (21 kDa), TRAIL-R2/DR5 (40 kDa), cFLIP (long isoform, 55 kDa), survivin (16 kDa), XIAP (53 kDa), Mcl-1 (40 kDa), Bcl-w (18 kDa), Puma (27 kDa) and Bad (23 kDa) were analysed by Western blotting. Analysis of β-actin (44 kDa) served as loading control. Largely similar results were obtained in three independent Western blot experiments using three independent series of cell extracts.

For illuminating celecoxib’s effects in cSCC cells, proteins involved in the control of apoptosis and cell proliferation were investigated in SCL-II and SCC-12 at 24 h of treatment by Western blotting. Suggesting a particular role in celecoxib-mediated inhibition of cell proliferation, the cell cycle inhibitor p21 was strongly upregulated by celecoxib in both cell lines. Further suggesting a particular role for TRAIL sensitivity, the major TRAIL receptor, DR5 (40 kDa), was significantly upregulated by celecoxib (Figure 7).

Several other proteins were suggestive for an enhancement of proapoptotic pathways by celecoxib. Thus, effector caspases may be blocked by cellular inhibitor of apoptosis proteins (cIAPs, e.g., survivin and chromosome X-linked inhibitor of apoptosis protein (XIAP)), whereas caspase-8 activity is suppressed by its competitive inhibitor cFLIP (cellular FLICE-like inhibitory protein). The long isoform of cFLIP (FLIP_L_, 55 kDa) was downregulated by celecoxib in both cell lines, and in SCL-II, also survivin and XIAP were downregulated either by celecoxib alone or by combination treatment (Figure 7).

The family of pro- and anti-apoptotic Bcl-2 proteins are critically involved in the control of intrinsic apoptosis pathways. Of the anti-apoptotic family members, Mcl-1 was downregulated in SCC-12 by celecoxib alone, whereas in SCL-II, both Mcl-1 and Bcl-w were downregulated by combination treatment. Also, the proapoptotic BH3-only family members, Puma and Bad, were upregulated in both cell lines by celecoxib alone (Figure 7), while for the anti-apoptotic Bcl-2 as well as for the proapoptotic Bax and Bak proteins, no significant changes in expression were seen (data not show). These findings underlined that celecoxib resulted in multiple effects on cell proliferation- and apoptosis-related proteins involved both in intrinsic and extrinsic apoptosis pathways.

### 2.6. Massive ROS Production by Celecoxib

**Figure 8:**
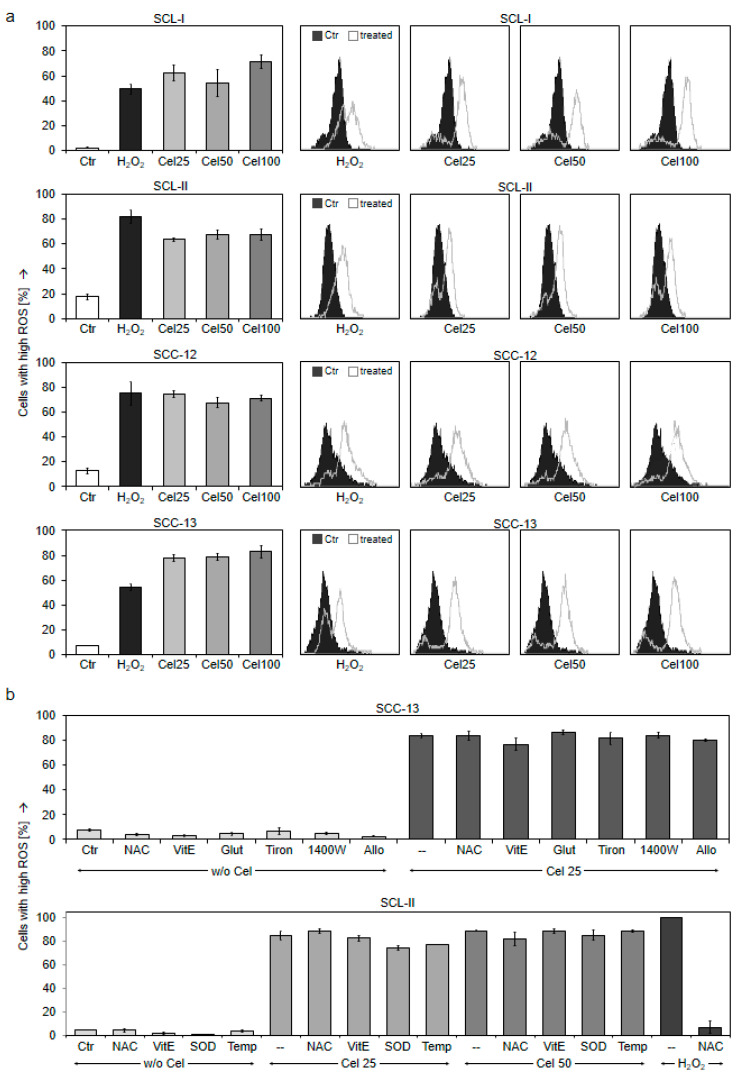
Strongly enhanced ROS levels by celecoxib. (**a**) Four cSCC cell lines were treated for 2 h with increasing concentrations of celecoxib (25, 50, and 100 µM), whereas H_2_O_2_ (1 mM, 1 h) was used as a positive control. ROS levels were determined by H_2_DCFDA staining and flow cytometry. Mean values +/− SDs of representative experiments are shown; at least two independent experiments, each one consisting of triplicate values, revealed highly comparable results. Example cytometry measurements (overlays vs. controls) are shown on the right side. (**b**) Treatment with different antioxidative strategies is shown in SCC-13 and SCL-II in addition to celecoxib treatment (25 and 50 µM): NAC, N-acetyl cysteine (1 mM); VitE, tocopherol (1 mM); Glut, glutathione (1 mM); Tiron (1 mM); 1400W (100 µM); Allo, Allopurinol (1 mM); Temp, Tempol (1 mM). Antioxydants were generally applied at 1 h before celecoxib.

Most impressive was the massive production of reactive oxygen species (ROS) in response to celecoxib, which appeared as an early effect already at 2 h in all cSCC cell lines. The vast majority of cells were responsive (54–75%) seen by an almost complete shift of the cells´ peak in flow cytometry. The full ROS shift was already obtained for the lowest celecoxib concentration of 25 µM, and the effects were even comparable to H_2_O_2_, used as a positive control (Figure 8a). Thus, high ROS production appeared as a typical result of celecoxib.

Increasing evidence in recent years has proven that ROS plays a vital role in the regulation of apoptosis. In previous work, in melanoma and cutaneous T-cell lymphoma cells, ROS production could often be prevented by antioxidant treatment as by N-acetyl cysteine (NAC) or by vitamin E (tocopherol), which thus allowed to prove the significant role of ROS in apoptosis [14,15]. This strategy, however, failed here for the high ROS levels produced in cSCC cells in response to celecoxib. Neither NAC nor tocopherol or several other antioxidative treatments tested were able to significantly prevent celecoxib-induced ROS production, as demonstrated for SCC-13 and SCL-II. In contrast, H_2_O_2_-induced ROS was decreased by NAC (Figure 8b). In agreement, apoptosis induced by celecoxib could not be reduced significantly by antioxidative pre-treatment (data not shown), suggesting that celecoxib-induced ROS was too strong.

## 3. Discussion

Cutaneous SCC and AK are characterized by high incidence and provoke severe health problems worldwide [1]. While many cSCCs can be treated by surgical excision and chemotherapy, a subset of patients develops therapy resistance, recurrence, and metastasis [23]. Furthermore, topical treatments of AK are often non-sufficient and cannot prevent tumor progression [24]. Thus, new and efficient treatments are needed for cSCC and AK.

The anti-inflammatory, analgesic, and anti-pyretic drug celecoxib has been in clinical use for many years [25]. Anti-neoplastic effects as inhibition of cancer cell growth and suppression of metastases have also been reported in vitro and in mouse models of colorectal, nasopharyngeal, and mammary carcinoma [26,27,28]. In clinical trials, celecoxib revealed positive results for colorectal and breast cancer [29], and it was approved for adjuvant treatment of familial adenomatous polyposis [17]. Another clinical trial suggested that progression from AK to cSCC may be diminished [30].

The efficiency of celecoxib may be improved in combinations, as shown, e.g., in non-Hodgkin lymphoma and melanoma cells with histone deacetylase inhibitors and plumbagin, respectively [31,32]. In clinical trials, improved response to chemotherapy was also obtained in combinations with celecoxib [33,34]. In the present study, celecoxib alone showed only little effects on apoptosis and cell viability in cultured cSCC cells, but apoptosis was strongly enhanced, and cell viability was decreased in combinations with death ligands. TRAIL represents a promising antitumor strategy due to its selective activity in cancer cells [9,10,11]. Thus, the efficiency of celecoxib may be crucially improved by combination with TRAIL or TRAIL receptor agonists.

Death ligands furthermore represent basic elements of an antitumor immune response driven by cytotoxic T-lymphocytes and natural killer cells [11]. Thus, enhancing death ligand-induced apoptosis by celecoxib may improve the immune response against cSCC. Positive combination effects of celecoxib and TRAIL were also found in other tumor cells, e.g., of non-small-cell lung carcinoma, colon cancer, and glioblastoma [35,36,37]. In contrast, in cSCC, the effects of TRAIL were not sufficiently investigated so far. Only for a combination of diclofenac and TRAIL an enhanced apoptotic response was shown [38].

A better understanding of the mechanisms of induced apoptosis can provide insights, which may be used for translational strategies. While not affected by celecoxib alone, proapoptotic caspase cascades were fully activated in combination with TRAIL, and their essential role was proven by a caspase inhibitor. Caspase dependency of celecoxib-induced apoptosis was also found in gastric cancer and non-small-cell lung cancer cells [39,40].

For demonstrating the decisive role of certain caspases, siRNA strategies may also apply. Thus, the role of caspase 8 in celecoxib-induced apoptosis in human lung cancer cells was shown [35], whereas apoptosis induced by another NSAID (FR122047) in MCF-7 breast cancer cells was even enhanced by siRNA knockdown of caspase-9 [41]. Due to the massive activation of caspases-3, -6, -7, -8, and -9 by celecoxib/TRAIL, we aimed to investigate the principle involvement of the caspase cascade, for which the used pan-caspase inhibitor appeared as particularly suitable.

Caspase-8 is antagonized by its competitive inhibitor cFLIP (cellular FLICE-like inhibitory protein), while cIAPs (cellular inhibitor of apoptosis proteins) as XIAP and survivin inhibit effector caspases and caspase-9 [42,43]. We show here the downregulation of cFLIP and survivin in cSCC cells in response to celecoxib, while XIAP was downregulated by the combination treatment in one cell line. Downregulation of cFLIP by celecoxib was also found in non-small-cell lung cancer cells [40], and it was downregulated in cSCC cells in response to diclofenac [38]. Roles of survivin and XIAP in celecoxib-mediated effects were also seen in leukemia and myeloma cells [44,45]. Downregulation of caspase antagonists thus appears as a frequent effect of celecoxib and may critically contribute to caspase activation in combinations.

In this study, highly pleiotropic effects of celecoxib were shown; namely, a number of investigated proteins were either upregulated or downregulated. Good examples in this context are upregulation of the cell cycle inhibitor p21 [46] and of the TRAIL receptor DR5, which may significantly affect inhibition of cell proliferation in cSCC cells and enhancement of TRAIL sensitivity, respectively. In colorectal cancer cells, p21 was also upregulated by celecoxib [47], and upregulation of DR5 by celecoxib was reported in non-small-cell lung cancer as well as in hepatocellular carcinoma cells [40,48].

Intrinsic, mitochondrial apoptosis pathways are critically controlled by Bcl-2 family proteins comprising of anti-apoptotic (e.g., Bcl-2, Mcl-1, and Bcl-w), proapoptotic multidomain (Bax and Bak) as well as proapoptotic BH3-only proteins (e.g., Puma and Bad) [6]. Suggesting an activation of intrinsic apoptosis pathways, Mcl-1 and Bcl-w were downregulated, while Puma and Bad were upregulated by celecoxib in cSCC cells. Mcl-1 is considered an important factor for the control of apoptosis in keratinocytes [49]. Its downregulation by celecoxib was also reported in hepatocellular carcinoma cells [50], and Bcl-w was downregulated in cSCC cells by diclofenac [51].

We have already previously shown the upregulation of Bad by celecoxib in the cell line SCL-II [51], and upregulation of Bad is shown here also in SCC-12. Upregulation of Puma by celecoxib was also found in stomach cancer cells, and its functional role in the activation of intrinsic apoptosis pathways was suggested [52]. In contrast, Puma was even downregulated by diclofenac in cSCC cells [51], thus suggesting a different mode of action in this setting.

The data of the present study suggest that celecoxib affects both intrinsic and extrinsic apoptosis pathways in cSCC cells. The question remains, how it can regulate so many different proteins. Here, reactive oxygen species (ROS) may serve as an explanation. ROS play important roles in tissue damage and aging, but in recent years an increasing body of evidence has also shown critical roles of ROS in apoptosis regulation in cancer cells. Thus in melanoma cells, apoptosis induction by different agents as an iron-substituted nucleoside analog, indirubin derivatives as well as by protein kinase B or BRAF inhibition was shown to be largely ROS-dependent [12,13,14,53]. Also in cutaneous T-cell lymphoma cells, apoptosis induction by indirubin derivatives was ROS-dependent [15]. Induction of ROS in the course of celecoxib treatment has already been reported in melanoma and breast cancer cells [54], and ROS production was suggested as a key mechanism for celecoxib´s anticancer activity in combination with 5-FU in head and neck cancer cells [55].

Here, we demonstrate massive ROS production by celecoxib in cSCC cells. ROS appeared very early and even at moderate celecoxib concentrations, suggesting ROS as upstream of other effects. While ROS were not sufficient to induce apoptosis themselves, they may prepare cSCC cells for apoptosis induction through death ligands. In previous studies, the significant role of ROS in apoptosis could be proven by antioxidative strategies, e.g., by N-acetyl cysteine or by tocopherol/vitamin E [12,13,14,53]. However, celecoxib-induced ROS in cSCC cells appeared as very strong and revealed characteristics, which could not be inhibited by different antioxidative strategies. Thus, the decisive role of ROS for celecoxib-induced apoptosis in cSCC cells is suggested here but could not be finally proven so far.

ROS levels in cells may also be regulated by antioxidative enzymes such as catalase and superoxide dismutase (SOD). For colon cancer, inhibition of tumor growth by celecoxib-loaded liposomes was accompanied by activation of SOD, which was further related to an antioxidative activity [56]. Celecoxib also increased SOD activity in rat stomach and colon mucosa [57]. In cSCC, however, the situation was clearly different, as ROS production by celecoxib in cSCC cells was strong, rapid, and long-lasting. We thus may not expect that CAT or SOD may be directly involved.

ROS induction appears as a promising anticancer strategy. Although the clinical effects of some ROS-inducing drugs were limited, as sulphasalazine in gastric cancer patients [58], other strategies have been favorably tested, e.g., the glutathione disulfide mimetic NOV-002 in patients with HER2-negative breast cancer [59]. Lack of efficiency may have many different causes, e.g., drug uptake, delivery, stability, and resistance mechanisms. Thus, limited efficiency of some strategies may not necessarily mean that ROS induction is not suitable for anticancer therapy.

At present, immune checkpoint inhibitors represent highly promising therapeutic strategies, also for cSCC. They may, however, not apply for all patients due to limitations because of side effects as well as therapy resistance [60]. Thus, new and additional anticancer strategies are still needed, which may also be used in combinations to improve efficiency. As shown here, celecoxib enhanced apoptosis sensitivity of cSCC cells for TRAIL. As TRAIL serves as an important factor of the immune system, it is suggestive that celecoxib in this way may also support an antitumor immune response. However, this effect could not be directly proven in the present study. Suitable animal experiments for investigating the function of the human immune response are rare and constitute a major challenge. Nevertheless, the antineoplastic effects of celecoxib have been shown in animal models of colorectal and mammary carcinoma [26,27].

In conclusion, lack of apoptosis induction by celecoxib alone suggests that its full therapeutic potential may be achieved only in combinations, and TRAIL appears as particularly suitable. As death ligands represent characteristic effectors of the immune system, celecoxib may also support an immune response against cSCC/AK, and combinations with immune checkpoint inhibitors recently approved for the treatment of cSCC may be considered. As concerning the mode of action in cSCC cells, highly pleiotropic effects on apoptosis regulators were found for celecoxib, which affects both intrinsic and extrinsic apoptosis pathways. ROS production by celecoxib appeared as most pronounced and may serve as a master regulator.

## 4. Materials and Methods

### 4.1. Cell Culture and Treatment

Four representative cSCC cell lines (SCL-I, SCL-II, SCC-12, and SCC-13) were used, which were derived from human facial skin lesions [38]. They were maintained at 5% CO_2_ in RPMI 1640 growth medium (Life Technologies, Darmstadt, Germany) supplemented with 10% FCS, 2 mM glutamine, non-essential amino acids, and antibiotics. Most assays were performed in 24-well plates after seeding 5 × 10^4^ cells per well. Treatments: celecoxib (BioMol, Hamburg, Germany; 1000-8672, 25–100 µM), agonistic anti-CD95 antibody (CH-11, IgM mouse; Beckman Coulter, Krefeld, Germany; 100 ng/mL), and Killer TRAIL^TM^ (Adipogen, San Diego USA, AG-40T-0001; 50 ng/mL). Control cells received the celecoxib´s solvent DMSO. For caspase inhibition, the pan-caspase inhibitor QVD-Oph (Abcam, Cambridge, UK; 10 µM) was given at 1 h before agonists were applied.

### 4.2. Cell Proliferation Assays

Growth rates were determined by real-time cell analysis (RTCA, xCELLigence; Agilent, Santa Clara, CA, USA). A number of 10,000 cells were seeded per well in special 96-well E-plates equipped with electrodes in the bottom of wells, and treatments started after 24 h. Electric resistance was continuously determined up to 140 h, which served as a measurement of cell confluence. A second cell proliferation assay was based on WST-1 staining (Water soluble tetrazolium-1, Roche Diagnostics), further analyzed by ELISA.

### 4.3. Determination of Apoptosis, Cytotoxicity and Cell Viability

Quantification of apoptosis was performed by cell cycle analysis. Cells were harvested by trypsinization and lysed in hypotonic buffer. Isolated nuclei were stained for 1 h with 40 mg/mL propidium iodide (Sigma-Aldrich, St. Louis, MO, USA). Cells in G1, G2, and S-phase, as well as sub-G1 cells, were quantified by flow cytometry at FL3A using a FACS Calibur (BD Bioscience, Bedford, MA, USA). Due to the washing out of small DNA fragments, nuclei with less DNA than G1 (sub-G1) correspond to apoptotic cells.

Cytotoxicity was determined in cell culture supernatants by quantification of released lactate dehydrogenase (LDH) activity. Cells were stained by a cytotoxicity detection assay (Roche Diagnostics, Penzberg, Germany) and analyzed by an ELISA reader. Triton x100-treated cells (0.7%) were used as a positive control.

Cell viability was determined by staining cells with calcein-AM (PromoCell, Heidelberg, Germany; AM = acetoxymethylester), which is converted in viable cells through intracellular esterase activity to green-fluorescent calcein. Cells, grown and treated in 24-well plates, were harvested by trypsinization and stained for 1 h with 2.5 µg/mL calcein-AM at 37 °C. After labeling, cells were washed with PBS and measured by flow cytometry (FL2H).

### 4.4. Mitochondrial Membrane Potential

Mitochondrial membrane potential (ΔΨm) was determined by staining cells with the fluorescent dye TMRM^+^ (Tetramethylrhodamin-methylester, Sigma-Aldrich). Cells, grown and treated in 24-well plates, were harvested by trypsinization and stained for 20 min at 37 °C with 1 µM TMRM^+^. After 2-times washing with PBS, staining of cells was quantified by flow cytometry (FL2H).

### 4.5. Analysis of Reactive Oxygen Species (ROS)

For determination of intracellular ROS levels, cells grown in 24-well plates were pre-incubated for 1 h with the fluorescent dye H_2_DCFDA (2’,7’-dichlorodihydrofluorescein diacetate, D-399, Thermo Fisher Scientific, Hennigsdorf, Germany, 10 µM), before starting treatment with effectors. After 2–24 h treatment, cells were harvested by trypsinization, washed several times with PBS, and analyzed by flow cytometry (FL1H). As a positive control, treatment with H_2_O_2_ (1 mM, 1 h) was applied. Different antioxidative treatments were used aiming at the suppression of celecoxib-induced ROS levels, including N-acetyl cysteine (NAC, Sigma-Aldrich, Taufkirchen, Germany, up to 4 mM), tocopherol (vitamin E, Fluka, Steinheim, Germany, up to 4 mM), glutathione (Sigma-Aldrich, 1 mM); Tiron (Sigma-Aldrich, 1 mM); 1400 W Sigma-Aldrich, 100 µM); Allopurinol (Sigma-Aldrich, 1 mM); TEMPOL (1-Oxyl-2,2,6,6-tetramethyl-4-hydroxypiperidine, Santa Cruz; 1 mM). Antioxidants were generally applied at 1 h before starting celecoxib treatment.

### 4.6. Western Blotting

For Western blotting, total protein extracts were obtained by cell lysis buffer containing 150 mM NaCl, EDTA (1 mM), 1% NP-40; 50 mM Tris (pH 8.0), as well as phosphatase and protease inhibitors. Following SDS polyacrylamide gel electrophoresis, proteins were blotted on nitrocellulose membranes.

Primary antibodies of Cell Signaling (Danvers, MA, USA): Caspase-3 (9662, rabbit, 1:1000), Cleaved caspase-3 (9664, rabbit, 1:1000), Caspase-8 (9746, mouse, 1:1000), Caspase-9 (9502, rabbit, 1:1000), Caspase-6 (9762, rabbit, 1:1000), Caspase-7 (9492, rabbit, 1:1000), XIAP (2042, rabbit, 1:1000), Mcl-1 (4572, rabbit, 1:1000), Bad (9292, rabbit, 1:1000), and Bcl-w (2724, rabbit, 1:1000). Primary antibodies of Santa Cruz Biotech (Dallas, TX, USA): c-FILP (sc-5276, mouse, 1:500), survivin (sc-177779, mouse, 1:500), p21(sc-6246, mouse, 1:500), p53 (sc-126, mouse, 1:500), β-actin (sc-47778, mouse, 1:1000), Puma (sc-374223, mouse, 1:500), Bax (sc-7480, mouse, 1:500), and Bak (sc-832, mouse, 1:500). Primary antibody of Abcam (Cambridge, UK): DR5 (ab8416, rabbit, 1:1000). Secondary antibodies: peroxidase-labelled goat anti-rabbit and goat anti-mouse (Dako, Hamburg, Germany; 1:5000).

### 4.7. Statistical Analyses

All assays were done in triplicate determinations, and at least two independent experiments were performed. Presented Western blot data were verified by at least two independent series of cellular extracts. Statistical significance was proven by Student’s *t*-test (two-tailed, heteroscedastic) using all data of independent experiments (at least six individual measurements); *p*-values < 0.05 were considered as statistically significant.
